# Dynein Function and Protein Clearance Changes in Tumor Cells Induced by a Kunitz-Type Molecule, Amblyomin-X

**DOI:** 10.1371/journal.pone.0111907

**Published:** 2014-12-05

**Authors:** Mario T. F. Pacheco, Carolina M. Berra, Kátia L. P. Morais, Juliana M. Sciani, Vania G. Branco, Rosemary V. Bosch, Ana M. Chudzinski-Tavassi

**Affiliations:** 1 Biochemistry and Biophysics Laboratory, Butantan Institute, São Paulo, Brazil; 2 Department of Biochemistry, Federal University of São Paulo, São Paulo, Brazil; University of South Florida College of Medicine, United States of America

## Abstract

Amblyomin-X is a Kunitz-type recombinant protein identified from the transcriptome of the salivary glands of the tick *Amblyomma cajennense* and has anti-coagulant and antitumoral activity. The supposed primary target of this molecule is the proteasome system. Herein, we elucidated intracellular events that are triggered by Amblyomin-X treatment in an attempt to provide new insight into how this serine protease inhibitor, acting on the proteasome, could be comparable with known proteasome inhibitors. The collective results showed aggresome formation after proteasome inhibition that appeared to occur via the non-exclusive ubiquitin pathway. Additionally, Amblyomin-X increased the expression of various chains of the molecular motor dynein in tumor cells, modulated specific ubiquitin linkage signaling and inhibited autophagy activation by modulating mTOR, LC3 and AMBRA1 with probable dynein involvement. Interestingly, one possible role for dynein in the mechanism of action of Amblyomin-X was in the apoptotic response and its crosstalk with autophagy, which involved the factor Bim; however, we observed no changes in the apoptotic response related to dynein in the experiments performed. The characteristics shared among Amblyomin-X and known proteasome inhibitors included NF-κB blockage and nascent polypeptide-dependent aggresome formation. Therefore, our study describes a Kunitz-type protein that acts on the proteasome to trigger distinct intracellular events compared to classic known proteasome inhibitors that are small-cell-permeable molecules. In investigating the experiments and literature on Amblyomin-X and the known proteasome inhibitors, we also found differences in the structures of the molecules, intracellular events, dynein involvement and tumor cell type effects. These findings also reveal a possible new target for Amblyomin-X, i.e., dynein, and may serve as a tool for investigating tumor cell death associated with proteasome inhibition.

## Introduction

Tumor cells exhibit several processes to maintain tumor mass as well as tumor cell proliferation, survival and metastasis capacity. These properties include the activation and inhibition of many different signaling pathways, such as death receptor suppression [1], nuclear factor-kappa B (NF-κB) activation [2], growth receptor signaling induction [3], procoagulant stimulation [4] and enhanced proteasomal activity [5], [6]. The ubiquitin-proteasome system (UPS) has emerged as a potential target for new chemotherapeutic agents [7]. The proteasome system has arisen as an important target due to its function in regulating a large number of cellular processes, such as the cell cycle [8] and the proteolysis of components of the NF-κB pathway [9].

The proteasome is a protein complex that is responsible for targeting major intracellular proteins for degradation [10]. However, the proteins targeted for degradation are marked with ubiquitin molecules by a series of specific enzyme reactions that involve one of ubiquitin's seven specific exposed lysine (K) residues; these residues are recognized by the proteasome [10]. One example is the K48 linkage of polyubiquitin, which targets protein substrates for UPS clearance [11]. Through K63 linkage, ubiquitin plays a role in aggresome formation, autophagy, endosomal trafficking, NF-κB signaling and DNA repair [11]. Thus, the cell has a specialized protein clearance flow that, in proteasome inhibition, induces the formation of protein aggregates that are organized into dynamic structures, aggresomes [11], that then activate autophagy [12]. High-molecular-weight protein aggregates can be excluded from the cell via this mechanism [12].

To eliminate the cytotoxic aggresomes after proteasome inhibition, these protein structures must be transported to a perinuclear region called the Microtubule Organizing Center (MTOC) by a molecular motor called dynein [12]. In the MTOC, the aggresomes are vesiculated by microtubule associated protein 1 light chain 3B (LC3B)-positive autophagosome membranes and fused with lysosomes for clearance via autophagy [13], [14]. There are two pathways in which dynein can transport aggresomes: (i) exclusive K63-polyubiquitinated protein aggregates linked to histone deacetylase 6 (HDAC6) [15] or (ii) the non-exclusive polyubiquitinated proteins mediated by the transfer of substrate from the chaperone heat shock protein 70 kDa (Hsp70) to BCl_2_-associated athanogene 3 (Bag3) [16]. Furthermore, despite its function in aggresome formation, dynein cytoplasmic 1 is the most abundant form among dyneins and is found in nearly all cells. Dynein cytoplasmic 1 is composed of one dimerized heavy chain (HC1), two intermediate chains (IC1 and IC2), two light-intermediate chains (LIC1 and LIC2) and six light chains (LC8-1, LC8-2, TcTex1, TcTex3, Roadblock1 and Roadblock2), which are all encoded by different genes [17]. This motor protein plays important roles beyond aggresome pathway function, for example in the transport of endosomes [18], autophagosomes and lysosomes [19], [20] and in the translocation of NF-κB dimer to the nucleus [21].

In this context, our group has been developing an antitumor molecule called Amblyomin-X (Ambly) [22]. The recombinant protein is a 15 kDa Kunitz-type serine protease inhibitor protein derived from a cDNA library construction of the salivary glands of the tick *Amblyomma cajennense* that has anti-coagulant properties [22], [23]. This molecule's Kunitz-type domain is similar to that of the endogenous tissue factor pathway inhibitor (TFPI) [22] and can reduce tumor growth and metastasis *in vivo* [23]. This protein also showed pro-apoptotic effects in tumor cells [23], [24], [25]. Another interesting aspect of the recombinant protein was its ability to upregulate the gene and protein expression of the dynein LIC2 chain in a microarray analysis and to preferentially inhibit the trypsin-like activity of the proteasome in tumor cells [24].

This study was intended to investigate common intracellular events linked to the supposed main target of Amblyomin-X (the proteasome) in the microenvironment of two different tumor cells: (i) human melanoma (SK-MEL-28) and (ii) human pancreas adenocarcinoma (MIA PaCa-2). Amblyomin-X exerted pro-apoptotic effects in both [24]. Some effects of Amblyomin-X were induced through its action on the proteasome; these effects were distinct from those of known proteasome inhibitors, as demonstrated by investigating intracellular protein quality control and dynein transport functions. However, some of the functions of Amblyomin-X related to aggresome formation and NF-κB function are shared by known proteasome inhibitors.

This study provides new insights into the molecular mechanism of action of Amblyomin-X, which is a promising candidate to treat tumor malignances. Our data provide points of comparison between this protein, which primarily acts in the proteasome, and known proteasome inhibitors that are small-cell-permeable molecules. This study also supports the utility of this multifaceted protein by investigating new strategies to enhance pharmacological effects in malignant cells.

## Materials and Methods

### Amblyomin-X preparation

The recombinant molecule was obtained as described elsewhere [22].

### Cell Culture

The MIA PaCa-2 and SK-MEL-28 human tumor cell lines were purchased from American Type Culture Collection (ATCC) and cultured as reported elsewhere [24].

### Antibodies

The following antibodies were used in this work: primary antibodies to β-actin, NFKB1, LC3B, and LIC2 and secondary antibody conjugated to horseradish peroxidase (HRP) (Abcam, Cambridge, UK); primary antibodies to HC1, LC8-1/2, mTOR, AMBRA1 and Bim (Santa Cruz Biotechnology Inc., Santa Cruz, CA); primary antibody to GAPDH (Sigma, St Louis, MO); K48-FITC and K63-Alexa Fluor 647 conjugated antibodies (Merck Millipore, Darmstadt, Germany); secondary antibodies to FITC, Alexa Fluor 647, Alexa Fluor 533 and Alexa Fluor 488, isotype control IgG1 conjugated with Alexa Fluor 647 and isotype control IgG1 conjugated with FITC were also purchased from Invitrogen Life Technologies Inc., USA.

### Cell viability assay

The 3-(4,5-dimethylthiazol-2-yl)-2,5-diphenyltetrazolium bromide (MTT) assay was used to measure the viability of AmblyominX-treated and non-treated cells and positive controls as described elsewhere [24].

### Gene expression

Gene expression was evaluated via quantitative real-time polymerase chain reaction (qPCR) using specific validated primers designed by the Primer Express 3.0 software (Applied Biosystems, Foster City, CA, USA) after the alignment of all isoforms to each target when needed.

The cells were cultured and treated, and the total RNA was extracted using an RNeasy Mini kit (Qiagen N.V. Netherlands) and quantified using a Nanodrop 2000 spectrophotometer (Thermo Scientific, USA). RNA integrity was verified via separation in a 2% agarose gel with 10× 3-(N-morpholino) propanesulfonic acid (MOPS), diethylpyrocarbonate (DPEC)-treated water and 37% formaldehyde and visualized using etidium bromide (1 mg/mL). Next, the RNA was treated with DNase I Amplification Grade kit (Invitrogen Life Technologies Inc., USA) and then used in first-strand cDNA synthesis via SuperScript III First-Strand Synthesis kit (Invitrogen Life Technologies Inc.). The cDNA was used in a SYBR green-based reaction with SYBR green Master Mix kit (Applied Biosystems, Foster City, CA, USA) in a Step One Plus thermal cycler (Applied Biosystems, Foster City, CA, USA). RNA levels were normalized to glyceraldehyde 3-phosphate dehydrogenase (GAPDH) and quantified using the Pfaffl method [26]. The melting curve was also verified for each target and negative control.

No positive controls, such as proteasome inhibitors, were used here due to the lack of information regarding their action on the gene expression of dynein chains and other targets related to intracellular protein quality control. The analysis compared Amblyomin-X-treated with non-treated cells.

### Western blotting analysis

Protein concentrations in whole-cell lysates were quantified via bicinchoninic acid (BCA) assay using a Pierce Microplate BCA Protein Assay kit (Thermo Scientific, USA). Protein expression was verified via separation in 7.5% (HC1), 10% (LIC2, mTOR, AMBRA1, β-actin and NFKB1) or 12.5% SDS-polyacrylamide gels (LC3, LC8-1/2 and Bim). The proteins were transferred to a polyvinylidene fluoride (PVDF) membrane. The samples were blocked with 5% bovine serum albumin (BSA) in tris-buffered saline with tween 20 (TBS-T) for 1 h and then incubated with the respective primary antibodies using GAPDH as an endogenous control followed by HRP secondary antibody incubation. Proteins were revealed via chemiluminescence with a homemade recipe (Tris 1.5 M pH 8.9, p-coumaric acid 20 mM, luminol 125 mM and hydrogen peroxide 30% in water).

Western blotting images were captured using an ImageQuant LAS 4000 (GE Healthcare, USA) and minimally processed (equally for all samples) using Windows Live software for Windows7 (Microsoft, Redmond, WA, USA). No positive controls, such as proteasome inhibitors, were used in the protein expression analysis due to the lack of information regarding their action on dynein. The analysis compared Amblyomin-X-treated with non-treated cells. The positive controls used for other targets were MG-132 and rapamycin (only for mTOR, AMBRA1 and LC3).

### Aggresome assay

Aggresome formation was visualized via fluorescence microscopy under an Olympus BX51 microscope with an Olympus XM10 camera (Olympus, Japan) and quantified via flow cytometry using a BD FACSCanto II cytometer (BD Biosciences, San Jose, CA, USA) following the instructions of the ProteoStat Aggresome Detection kit (Enzo Life Science Inc., Farmingdale, NY, USA). According to the commercial kit used, aggresome formation is declared when the arbitrary units exceed by 25 units, such as the provided proteasome inhibitor, MG-132 do.

Aggresomes were characterized using transmission electron microscopy. The cells were cultured in 25 cm^2^ flasks and collected with a scraper following drug incubation. The cells were gently harvested and fixed with 2.5% glutaraldehyde for 3 h at room temperature followed by 4°C. The samples were then postfixed with 1% osmium tetroxide for 2 h at 4°C. The cells were stained with 2% aqueous uranyl acetate for 2 h at 4°C in the dark, dehydrated through a series of acetone and propylene oxide and then embedded in epoxy resin with methanol (1∶1). The resin was sectioned, and the samples were analyzed in an LEO 906 E transmission electron microscopy (Zeiss, Germany) and photographed with a Mega View III camera (Zeiss, Germany) with ITEM Olympus Soft Imaging Solutions software. MG-132 was used as the positive control.

Aggresome signaling was analyzed by quantifying intracellular HDAC6 and Bag3 protein concentration via enzyme-linked immuno sorbent assay (ELISA) with HDAC6 ELISA and Bag3 ELISA kits (USCN Life Science Inc., Wuhan, China) according to the manufacturer's instructions. No positive controls, such as proteasome inhibitors, were used due to the lack of information regarding their action at these protein levels. The analysis compared Amblyomin-X-treated and non-treated cells.

### Autophagy assay

Immunofluorescence was measured using a specific antibody to LC3B-positive autophagosome vesicles. Cells were grown on sterile coverslips and fixed with 4% paraformaldehyde for 15 min at room temperature, washed and permeabilized with 0.5% Triton X-100/0.6% EDTA 0.5 M pH 8.0 for 15 min at room temperature. The samples were blocked with 1% BSA for 30 min at room temperature and then incubated with primary antibody overnight at 4°C. The cells were then incubated with secondary antibody for 1 h at room temperature in the dark, and the coverslips were removed from the plate and mounted on glass slides with one drop of Vecta Shield anti-fade reagent (Vecta Labs, Burlingame, CA, USA). Western blotting was also employed to verify LC3 conversion (I to II) as described above. The secondary antibody was tested alone to verify that it did not autofluoresce. MG-132 and rapamycin were used as positive controls.

An acidic vesicle assay was also employed. This method was based on non-specific acridine orange stain, which exhibits bright green fluorescence in the cytoplasm and nucleus and bright red fluorescence in acidic vesicles, such as lysosomes. The cells were grown on coverslips and incubated with acridine orange (1 µg/mL) in the dark for 15 min at room temperature and then fixed with 4% paraformaldehyde for 20 min in the dark at room temperature. The coverslips were mounted in the same manner, and the samples were analyzed under a Zeiss LSMS10 fluorescence microscope (Zeiss, Germany) under 488 nm laser irradiation and acquired using Zeiss LSMS10 3.2.1 software (Zeiss, Germany). MG-132 and rapamycin were used as positive controls.

### Ubiquitin signaling

The cells were cultured and collected in cytometer tubes with trypsin/EDTA (0.25%/0.53 M) and incubated in 4% paraformaldehyde for 30 min on ice. The samples were then washed and ressuspended in permeabilizing solution (0.5% Triton-X 100/0.6% EDTA 0.5 M pH8.0) and incubated for 30 min on ice. The samples were washed and incubated with anti-K48 or anti-K63 antibody for 45 min on ice and then analyzed in a FACS Cantho II flow cytometer (BD Biosciences, San Jose, CA, USA) using FACS Diva 6.3.1 software (BD Biosciences). The data were analyzed in FlowJo (Trestar, CA, USA), and graph bars were constructed based on the mean fluorescence intensity obtained from histograms. No positive controls, such as proteasome inhibitors, were used due to the lack of information regarding their action at these ubiquitin levels. The analysis compared Amblyomin-X-treated and non-treated cells.

### Confocal microscopy

Cells were grown on sterile coverslips, fixed with 4% paraformaldehyde for 15 min at room temperature, washed and permeabilized with 0.5% Triton X-100/0.6% EDTA 0.5 M pH 8.0 for 15 min at room temperature. The samples were blocked with 1% BSA for 30 min at room temperature and then incubated with primary antibodies overnight at 4°C. The cells were then incubated with secondary antibodies for 1 h at room temperature in the dark, and the coverslips were removed from the plate and mounted in glass slides with one drop of Vecta Shield anti-fade reagent (Vecta Labs, Burlingame, CA, USA). The images were analyzed with a Zeiss LSMS 510 confocal microscope (Zeiss, Germany) and LSM Image software (Zeiss, Germany). No positive controls, such as proteasome inhibitors, were used due to the lack of information regarding their action on dynein-target interaction. The analysis compared Amblyomin-X-treated and non-treated cells. The secondary antibodies were verified to lack autofluorescence.

### Statistical analysis

Inference studies were carried out using two-way ANOVA analysis followed by Bonferroni's post-hoc test in GraphPad Prism 5.0 software (GraphPad Software Inc., San Diego, CA). Statistical significance was set at p≤0.05.

## Results

### Amblyomin-X induces different gene expression of dynein and targets related to protein quality control between tumor cell lines

**Figure 1:**
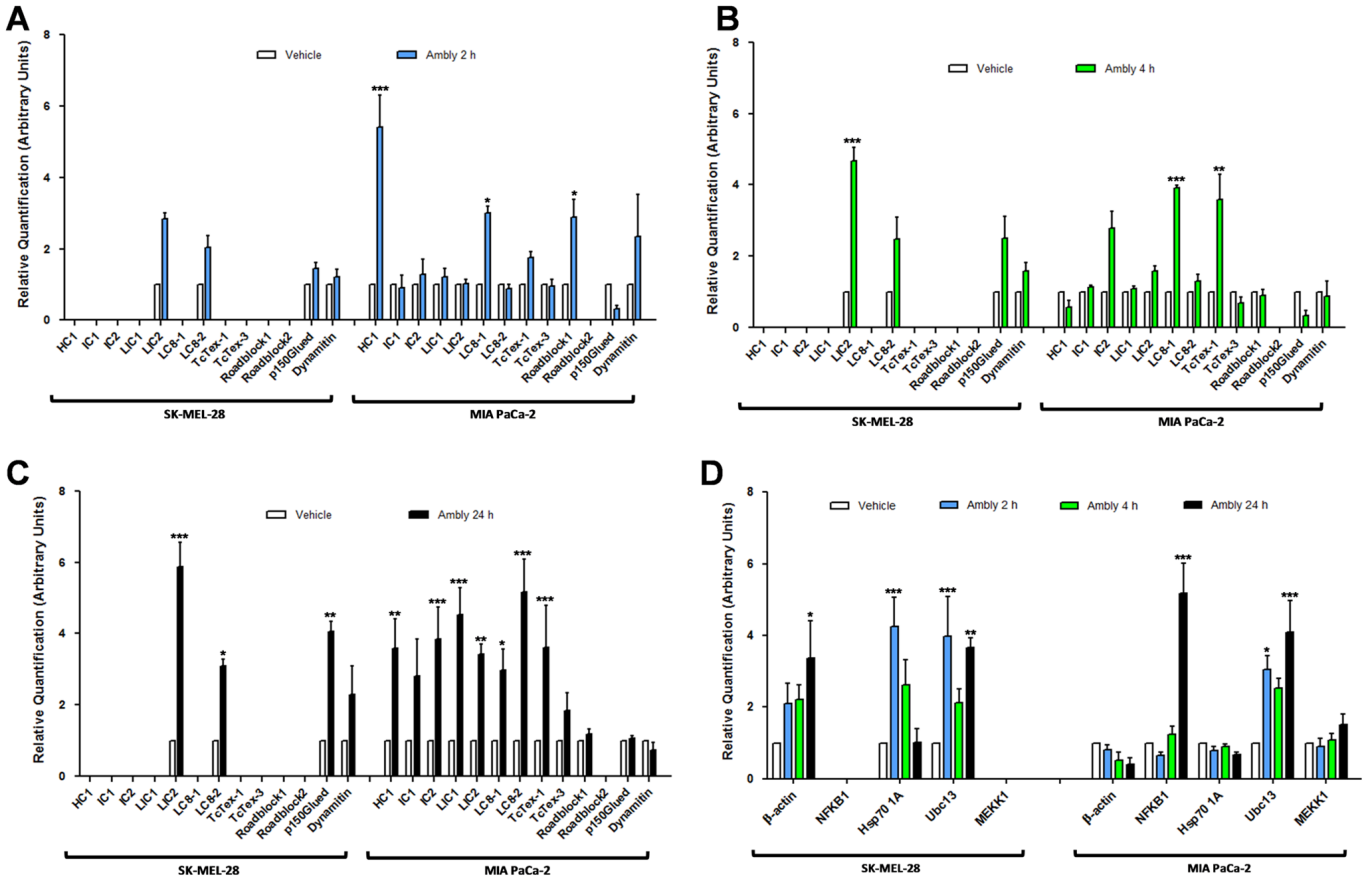
Gene expression of dynein and targets related to intracellular protein quality control induced by Amblyomin-X. qPCR analysis of dynein chains and two chains of dynactin (p150Glued and dynamitin) with Ambly induction (**A**) 2 h, (**B**) 4 h and (**C**) 24 h. qPCR analysis of targets related to intracellular protein quality control with Ambly induction (**D**) for 2 h, 4 h and 24 h. Cultured cells were treated with vehicle (phosphate buffered saline, PBS) or 0.5 µM Ambly for 2 h, 4 h or 24 h. The results were calculated related to the control (vehicle) and are expressed as the means ± standard error of fold increase over control (considered as 1) in arbitrary units. Three independent experiments were performed. The criteria and representation of statistical significance were set as *p≤0.05, **p≤0.01, ***p≤0.001 or ns (non-significant).

To explore other dynein-related changes beyond the gene overexpression of the dynein LIC2 chain observed via microarray analysis [24], we investigated other dynein chains using qPCR. In SK-MEL-28 cells, only dynein LIC2 and LC8-2 and two components of a dynein regulator, dynactin (p150Glued and dynamitin) [27], [30], could be assessed using this method due to a constitutively low amplification of the other chains (**Fig. 1A, 1B and 1C**). We observed increased mRNA expression of LIC2 after 4 h and increased LIC2, LC8-2 and p150Glued after 24 h of Amblyomin-X treatment (**Fig. 1B and 1C**). In MIA PaCa-2 cells, the only chain that we could not analyze was Roadblock2 (**Fig. 1A, 1B and 1C**). In this cell type, we observed an increased mRNA expression of some chains after 2 h and 4 h (**Fig. 1A and 1B**) and most of the chains after 24 h of Amblyomin-X treatment (**Fig. 1C**); however, no changes were observed in dynactin components after any treatment duration (**Fig. 1A, 1B and 1C**). The amount of cDNA template was increased in the targets with low amplification; however, this increase was not sufficient to calculate the mRNA levels (or the GAPDH curve was displaced too far to the left), thus making the gene expression calculation impossible (data not shown).

We next examined the mRNA levels of targets related to intracellular protein quality control: the chaperone Hsp70, which is involved in the non-exclusive ubiquitin aggresome formation pathway [28]; the E2-conjugating K63-specific enzyme Ubc13 [29]; and the mitogen-activated protein kinase (MAPK) MEKK1, which is involved in aggresome particle recruitment without requiring its kinase function [11]. The most abundant subunit of the transcription factor NF-κB (NFKB1) acts as a dimer (primarily with the RelA subunit) [31]. The proteasome inhibitors already described, reduce the transcriptional function of the NF-κB [32]. Therefore, NFKB1 was also analyzed to evaluate NF-κB activity in Amblyomin-X-treated cells. Finally, β-actin was used as a target because preliminary tests revealed mRNA changes in the tumor cell lines after Amblyomin-X treatment (data not shown).

The qPCR analysis showed that neither MEKK1 nor NFKB1 could be assessed using this method in SK-MEL-28 cells (**Fig. 1D**). However, β-actin and Ubc13 were upregulated in this cell line, especially after 24 h of Amblyomin-X treatment (**Fig. 1D**). Hsp70 and Ubc13 were changed after 2 h (**Fig. 1D**). In MIA PaCa-2 cells, only Ubc13 and NFKB1 were increased, especially after 24 h of treatment with the recombinant protein (**Fig. 1D**). Ubc13 was upregulated starting at 2 h (**Fig. 1D**).

### Dynein, NFKB1 and β-actin protein expression changes induced by Amblyomin-X

Next, we investigated protein expression levels via a western blotting analysis of dynein. Three chains were analyzed: (i) HC1, as this protein is the ATP-dependent molecular motor that anchors the intermediate and light intermediate chains [20]; (ii) LIC2, which was overexpressed in microarray analysis [24] and in qPCR experiments; and (iii) LC8-1 and LC8-2 because we observed changes in LC8-2 mRNA in both tumor cell lines and in LC8-1 mRNA in MIA PaCa-2 cells (**Fig. 1A, 1B and 1C**). The NFKB1 subunit of the NF-κB transcription factor and β-actin were also evaluated.

**Figure 2:**
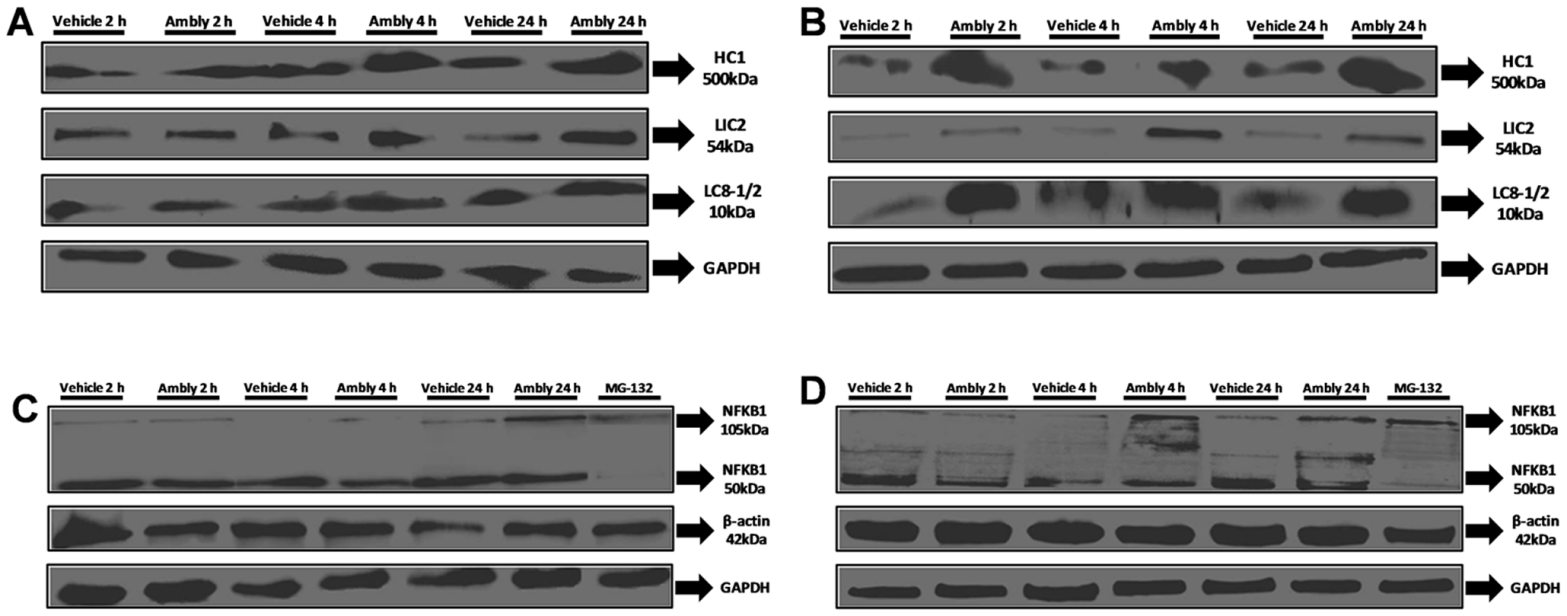
Protein expression of dynein, NFKB1 and β-actin induced by Amblyomin-X. Representative western blots of whole-cell lysates of (**A**) dynein chains in SK-MEL-28 cells, (**B**) dynein chains in MIA PaCa-2 cells, (**C**) NFKB1 and β-actin in SK-MEL-28 cells and (**D**) NFKB1 and β-actin in MIA PaCa-2 cells. Cultured cells were treated with vehicle (PBS), 0.5 µM Ambly for 2 h, 4 h or 24 h or 5 µM MG-132 (NFKB1 and β-actin). Images are representative of three independent experiments.

Both tumor cell lines treated with Amblyomin-X showed increased HC1 after 2 h, 4 h and 24 h (**Fig. 2A and 2B**). Additionally, LIC2 was preferentially overexpressed after 4 h and 24 h (**Fig. 2A and 2B**) and LC8-1/2 after 2 h, 4 h and 24 h (**Fig. 2A and 2B**).

Interestingly, although we could not quantify NFKB1 gene expression in SK-MEL-28 cells via qPCR (**Fig. 1D**), the two tumor cell lines treated with Amblyomin-X showed a block in the proteolysis of the NFKB1 subunit p105 after 24 h of treatment (**Fig. 2C**).

Amblyomin-X treatment in MIA PaCa-2 cells also showed increased NFKB1 subunit protein expression after 4 h and 24 h reflecting a block of the proteolysis of p105 into active p50 (**Fig. 2D**).

Finally, we noticed that in SK-MEL-28 cells, β-actin slightly increased after 24 h of Amblyomin-X treatment (**Fig. 2C**), which was not observed in MIA PaCa-2 cells (**Fig. 2D**).

### Aggresome formation induced by Amblyomin-X after proteasome inhibition

In previous studies, our group demonstrated the inhibition of both the trypsin- and chymotrypsin-like activities of the proteasome in MIA PaCa-2 and SK-MEL-28 cells after 24 h of Amblyomin-X treatment [24]. Thus, in this work, we aimed to visualize and quantify the aggresome formation induced by Amblyomin-X alone and by inhibiting protein synthesis with cycloheximide (CHX) [33]. We also used a known proteasome inhibitor, MG-132, as a positive control because proteasome inhibitors are expected to induce aggresome formation [11].

**Figure 3:**
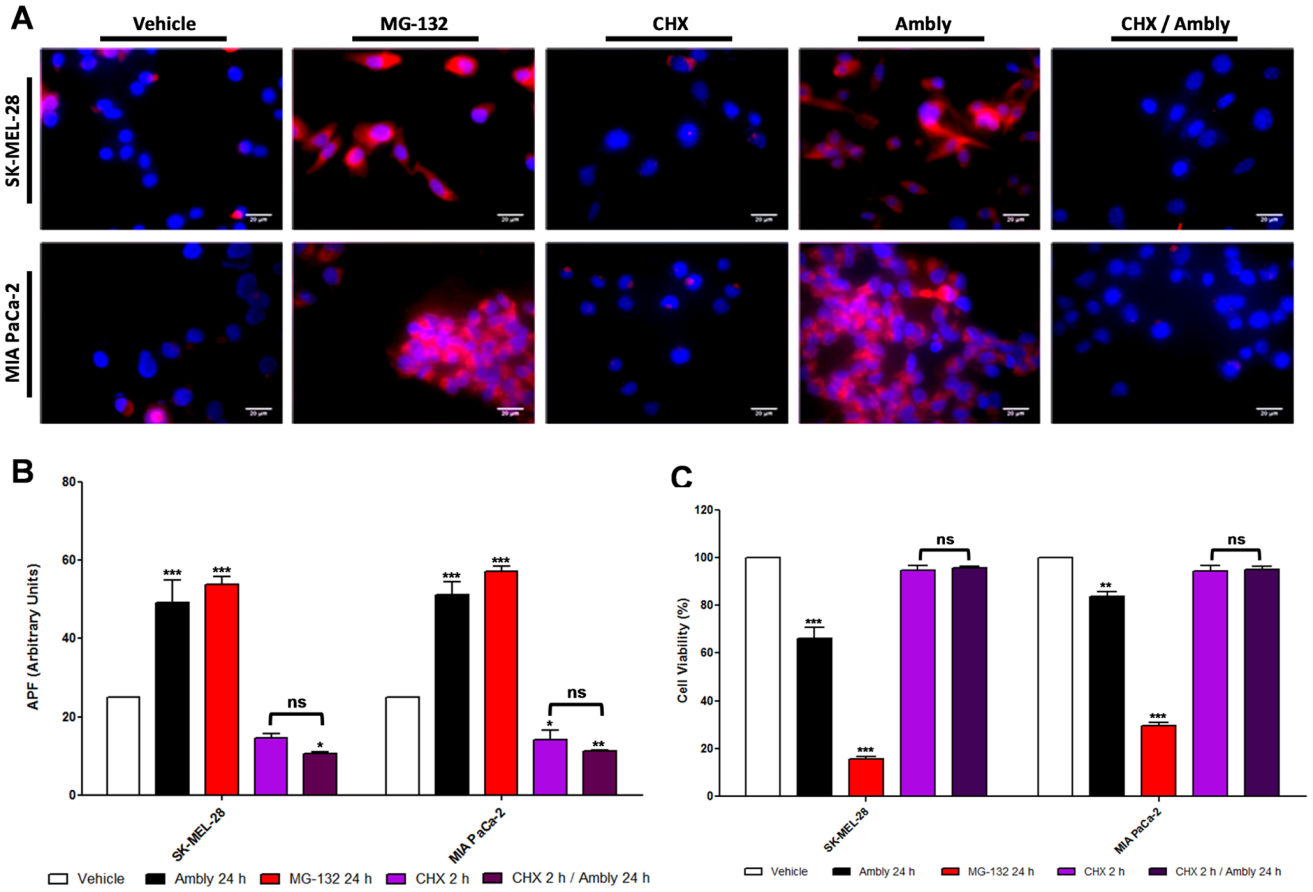
Aggresome formation induced by Amblyomin-X. Cultured cells were treated with vehicle (PBS), 0.5 µM Ambly for 24 h, 5 µM MG-132 for 24 h, 3.5 µM CHX for 2 h or 0.5 µM Ambly for 24 h after pretreatment with 3.5 µM CHX for 2 h (CHX/Ambly). (**A**) Fluorescence microscopy analysis of aggresomes. Aggresomes were labeled with a commercial kit in red and nuclei were stained with Hoechst 33342 in blue. Images are representative of five fields from each experiment (n = 3). (**B**) Mean fluorescence intensity obtained from histograms of flow cytometry analysis of aggresomes. Results are reported as the means ± standard error of agressome propensity factor (APF) in arbitrary units calculated according to the manufacturer's instructions. Three independent experiments were performed. The criteria and representation of statistical significance were set as *p≤0.05, **p≤0.01, ***p≤0.001 or ns (non-significant). (**C**) Cell viability assay of tumor cells treated with the compounds used in the aggresome analysis. Results are reported as the means ± standard error of three independent experiments. The criteria and representation of statistical significance were set as *p≤0.05, **p≤0.01, ***p≤0.001 or ns (non-significant).

Our data showed aggresome formation after proteasome inhibition induced by the recombinant protein treatment in both tumor cells lines (**Fig. 3A and 3B**). Aggresome formation was also blocked when the cells were pretreated with CHX (**Fig. 3A and 3B**). Cell viability was decreased in both tumor cell lines only when treated with Amblyomin-X alone or with MG-132 (**Fig. 3C**).

**Figure 4:**
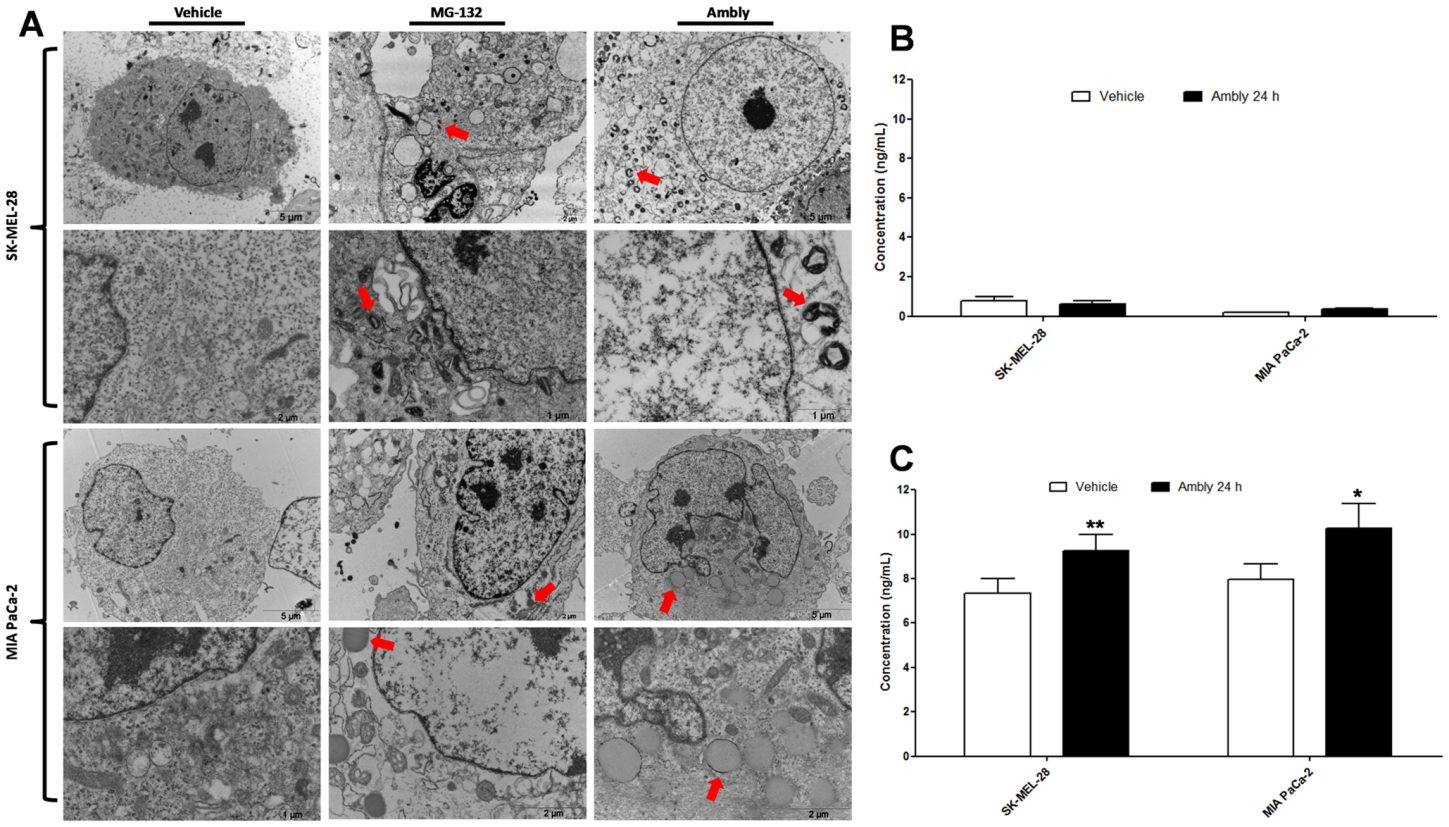
Characterization of aggresomes induced by Amblyomin-X. (**A**) Transmission electron microscopy analysis. Cultured cells were treated with vehicle (PBS), 0.5 µM Ambly for 24 h or 5 µM MG-132 for 24 h. Formed aggresomes are indicated by red arrows. Images are representative of five fields from each experiment (n = 3). Quantification of (**B**) HDAC6 and (**C**) Bag3 via ELISA. Cultured cells were treated with vehicle (PBS) or 0.5 µM Ambly for 24 h. Results are reported as the means ± standard error of three independent experiments. The criteria and representation of statistical significance were set as *p≤0.05, **p≤0.01, ***p≤0.001 or ns (non-significant).

Using transmission electron microscopy, we next characterized the type of aggresomes that formed. Aggresomes occur in two forms: spherical and ribbon-like [11]. Both types can be composed of different substrates, such as multispanning transmembrane proteins, secretory proteins and cytosolic proteins [11]. The structure varies depending on the aggregating substrate and the cell type [11]. Our results showed that Amblyomin-X induced spherical-type aggresome formation in MIA PaCa-2 cells (**Fig. 4A**), and ribbon-type formation in SK-Mel-28 cells (**Fig. 4A**) as did MG-132 (**Fig. 4A**).

To investigate which pathway of aggresome formation is involved in the Amblyomin-X mechanism of action, we assessed the intracellular protein levels of both HDAC6 and Bag3 using ELISA. HDAC6 did not change after Amblyomin-X treatment (**Fig. 4B**), but Bag3 increased; this occurred in both tumor cell lines (**Fig. 4C**).

### Ubiquitin profile and autophagy dysfunction induced by Amblyomin-X

Amblyomin-X induces the accumulation of polyubiquitinated proteins after proteasome inhibition [24]. Due to the results of linkage studies on ubiquitin K48 and K63 (which associated aggresome formation with other cell aspects), we performed a flow cytometry analysis using specific conjugated antibodies against polyubiquitinated K48 and K63 to identify the polyubiquitin profile after Amblyomin-X treatment.

**Figure 5:**
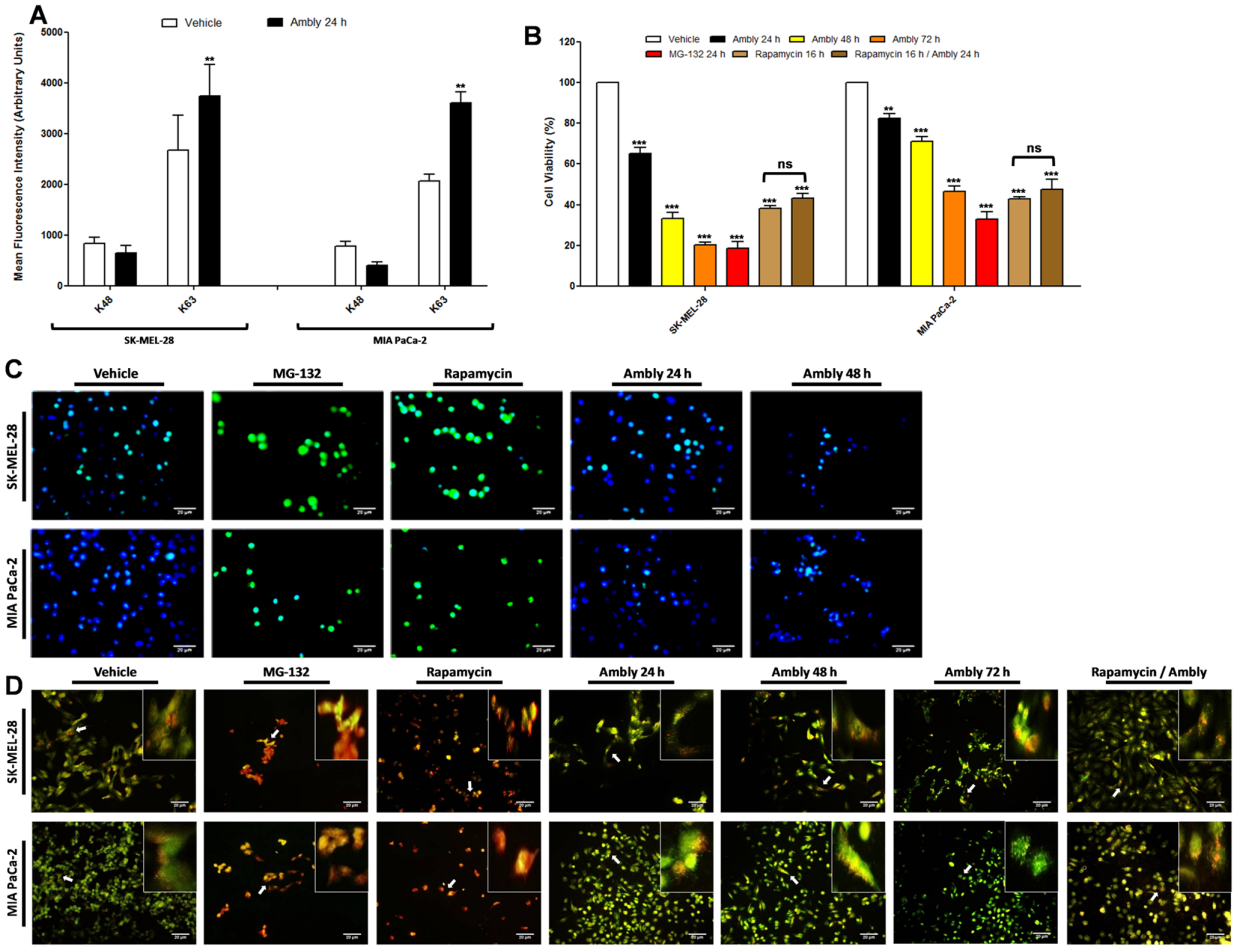
K-linkage profile and visualization of autophagy steps in the mechanism of action of Amblyomin-X. (**A**) Mean fluorescence intensity obtained from histograms of flow cytometry analysis of K48 and K63 linkage. Cultured cells were treated with vehicle (PBS) or 0.5 µM Ambly for 24 h. Results are expressed as the means ± standard error in arbitrary units of three independent experiments. The criteria and representation of statistical significance were set as *p≤0.05, **p≤0.01, ***p≤0.001 or ns (non-significant). (**B**) Immunofluorescence analysis of autophagic membrane formation. Cultured cells were treated with vehicle (PBS), 5 µM MG-132 for 24 h, 0.2 µM rapamycin for 16 h or 0.5 µM Ambly for 24 h or 48 h. LC3 was stained with FITC and is represented in diffused green fluorescence in the cytoplasm, while the nucleus was stained with DAPI and is represented in blue. Images are representative of five fields from each experiment (n = 3). (**C**) Fluorescence microscopy analysis using acridine orange stain. Cultured cells were treated with vehicle (PBS), 5 µM MG-132 for 24 h, 0.2 µM rapamycin for 16 h, 0.5 µM Ambly for 24 h pretreated with 0.2 µM rapamycin for 16 h (rapa/Ambly) or 0.5 µM Ambly for 24 h, 48 h or 72 h. Bright red fluorescence indicates acidic vesicles, while green fluorescence indicates the cytoplasm and nucleus. White arrows indicate the zoomed area located in the upper right position of the image. Images are representative of five fields from each experiment (n = 3). (**D**) Cell viability assay of tumor cells treated with the compounds used in autophagy visualization. Results are reported as the means ± standard error of three independent experiments. The criteria and representation of statistical significance were set as *p≤0.05, **p≤0.01, ***p≤0.001 or ns (non-significant).

The K48 profile did not change in either tumor cell line after treatment with the recombinant molecule (**Fig. 5A**). Interestingly, both tumor cell types treated with Amblyomin-X had increased polyubiquitinated K63 proteins (**Fig. 5A**).

Proteasome inhibitors are expected to induce autophagy [32]; thus, our next investigation was performed in three steps. First, we performed immunofluorescence experiments labeling LC3 with a specific antibody and secondary FITC antibody to verify autophagic membrane formation. This can be visualized via diffuse fluorescence throughout the cytoplasm. The findings revealed that Amblyomin-X did not induce diffuse fluorescence in either of the tumor cell lines even after 48 h of treatment (**Fig. 5B**). The same as observed with the positive controls [the proteasome inhibitor MG-132 and the inhibitor of mTOR rapamycin] [41] (**Fig. 5B**).

The second assay was performed to analyze the autophagy pathway. Autophagy vesicles are acidic [34]; we verified the formation of acidic vesicles using acridine orange. This is a non-specific compound that becomes protonated in acidic vesicles, to form aggregates that exhibit bright red fluorescence, whereas the cytoplasm and nucleus show dominant green fluorescence [34]. The results showed that Amblyomin-X did not induce an increase in red-labeled acidic vesicles in either of the cell lines analyzed (in contrast to the positive controls) even at 48 h and 72 h after Amblyomin-X treatment (**Fig. 5C**). Interestingly, both of the cell lines that were pretreated with rapamycin and then treated with Amblyomin-X acidic vesicle normalization (**Fig. 5C**).

We also performed a cell viability assay, which showed decreased viability in both tumor cell lines when treated with Amblyomin-X (24 h, 48 h and 72 h), MG-132, rapamycin and rapamycin/Ambly (**Fig. 5D**). Our data showed no differences in cell viability between treatment with rapamycin and rapamycin/Ambly (**Fig. 5D**).

**Figure 6:**
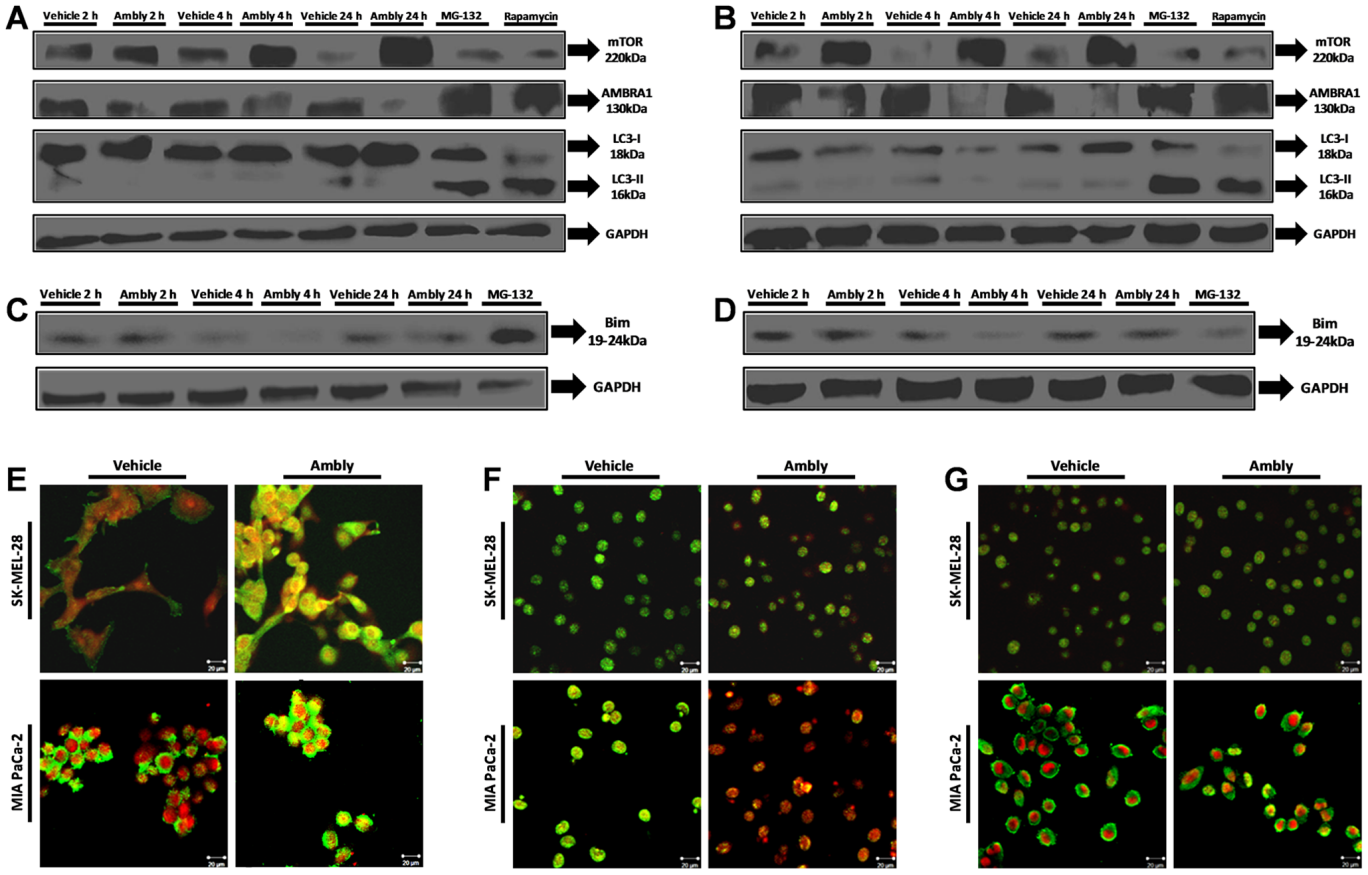
Relationship between autophagy marker expression and dynein in the mechanism of action of Amblyomin-X. Representative western blots of whole-cell lysates of cultured cells treated with vehicle (PBS), 0.5 µM Ambly for 2 h, 4 h or 24 h, 5 µM MG-132 for 24 h or 0.2 µM rapamycin for 16 h. Images are representative of three independent experiments containing the autophagic markers (mTOR, AMBRA1, LC3-I and LC3-II) in **A**) SK-MEL-28 cells and (**B**) MIA PaCa-2 cells and (**C**) autophagic/apoptosis marker (Bim) in SK-MEL-28 cells and (**D**) MIA PaCa-2 cells. Confocal microscopy analysis of cultured cells treated with vehicle (PBS) or 0.5 µM Ambly for 24 h. The final overlay image represents five fields of three independent experiments in which (**E**) the red fluorescence represents HC1, while the green fluorescence represents mTOR and the merging of the two is in yellow; or (**F**) the red fluorescence represents LC8-1/2, while the green fluorescence represents AMBRA1 (originally, the yellow fluorescence was artificially colored by the microscope software) and the merging of the two is in yellow; or (**G**) the red fluorescence represents LC8-1/2, while the green fluorescence represents Bim and the merging of the two is in yellow.

To confirm these results, we used a western blotting analysis of LC3 to verify the conversion of LC3-I into LC3-II, which would indicate autophagic membrane formation [13], [14]. The recombinant protein could not induce conversion of the molecule after 24 h of treatment in either of the cell lines studied (in contrast to the positive control). (**Fig. 6A and 6B**).

Because mammalian target of rapamycin (mTOR) works as a negative regulator of autophagy [42], we decided to evaluate its protein expression. We observed that Amblyomin-X induced the overexpression of mTOR after 2 h, 4 h and 24 h of treatment (**Fig. 6A and 6B**). We also measured activating molecule in Beclin-1-regulated autophagy (AMBRA1). This target is an activator of autophagy but is inactive when bound to dynein [38], [43]. Interestingly, Amblyomin-X decreased protein expression of AMBRA1 after 2 h, 4 h and 24 h of treatment in both cell lines (**Fig. 6A and 6B**).

We finally investigated the pro-apoptotic factor Bcl-2-like protein 11 (Bim), which is inactive when bound to dynein [38], [43] and can act as an inhibitor of autophagy by recruiting coiled-coil myosin-like BCL-2 interacting protein (Beclin-1) and consequently AMBRA1 to dynein [39], [45]. We observed that Amblyomin-X induced no changes in the protein expression of this target in either tumor cell line (**Fig. 6C and 6D**).

Our next step was to investigate the co-localization of dynein chains to those targets. First, we analyzed the HC1/mTOR interaction because dynein participates in mTOR co-localization [44]; we observed an increased co-localization of the targets in both tumor cell lines (**Fig. 6E**). Second, the interaction between LC8-1/2 and AMBRA1 was also investigated; we found that Amblyomin-X induced the co-localization of these two proteins only in MIA PaCa-2 cells (**Fig. 6F**). Finally, we analyzed the interaction between LC8-1/2 and Bim and observed no changes in their localization in either tumor cell line (**Fig. 6G**).

## Discussion

This study describes a novel Kunitz-type protein of approximately 15 kDa [22] that primarily acts on proteasomes. Amblyomin-X exhibits some features that differentiate this protein from other known proteasome inhibitors. We previously described its action in a variety of tumor cells [23], [24], [25]. Amblyomin-X inhibits proteasome activity as early as 4 h and for as long as 24 h, primarily by inhibiting the trypsin-like activity of the proteasome [24]. The proteasome inhibitors described preferentially inhibit chymotrypsin-like activity [7], [46] and are all small molecules or peptides [32]; thus, they pass directly through the plasma membranes of cells. Bortezomib [35] and carlfizomib [36], which are approved by the U.S Food and Drug Administration (FDA), are not involved with any specialized uptake mechanism by the cell or a special recognition by the tumor cell membrane. Amblyomin-X uptake by tumor cells and the involvement of dynein are under investigation. Amblyomin-X is a relatively large protein; thus, the current hypothesis is that this protein may require a specialized uptake mechanism such as endocytosis.

Proteasome inhibitors, such as carlfizomib, exhibit a selectivity strictly related to their action on the catalytic subunit of the proteasome [7]; thus, they do not act on other proteases as bortezomib does [46]. In addition to this, proteasome inhibitor use is limited to therapies for multiple myeloma and blood malignancies [7], [46]. In this study, we explored a new molecule that acts on the proteasome in two tumor cell lines representing solid tumors (SK-MEL-28 and MIA PaCa-2). We previously reported the pro-apoptotic effects of Amblyomin-X in both cell lines [24].

In this context, we investigated the intracellular events that could be used to distinguish Amblyomin-X from other compounds that target the UPS. Interestingly, the recombinant protein presence in the tumor cell microenvironment appears to trigger a signal transduction that leads to an increased gene expression profile of particular dynein chains that showed some differences between the tumor cell lines studied. Some analyzed targets could not be quantified using qPCR, especially in SK-MEL-28 cells, possibly because of the presence of a great number of alternative splicing mRNA variants in these cells; tumor cells produce many mutant proteins [37]. Another hypothesis is that the pair of primers did not target the sequence site most suited to properly access the amplification of the target. However, the primers were validated with good efficiency, the RNA sample quality was verified via agarose gel electrophoresis and PCRs were performed with endogenous controls. Some genes of interest may not be constitutively expressed at a high level in a specific cell type due to the increased cDNA sample and similar test results (data not shown).

The ATP-dependent HC1 motor chain of dynein is responsible for the movement of this complex and the transportation of cargoes along microtubules to their destinations [20]. Although HC1 gene expression could not be measured in SK-MEL-28 cells after Amblyomin-X treatment, both tumor cell types displayed an increased protein expression of this motor chain. In addition to this, LIC2 and LC8-1/2 protein expression were increased after Amblyomin-X induction.

Proteasome inhibition leads to aggresome formation [11] and requires dynein function because this molecular motor transports aggresomes [11]. Additionally, K63 signaling encompasses aggresome formation, autophagy activation, endosome trafficking, NF-kB and DNA repair signaling [11]. The data from this study showed increased gene and protein expressions of dynein chains in addition to increased K63 linkage, suggesting a possible role for dynein in other cellular functions in the Amblyomin-X mechanism of action beyond the expected transport of aggresomes.

Dynein is also a binding partner of the main heterodimer of NF-κB (NFKB1 (p105/p50)/RelA (p65)) [21]. In the tumor cells studied, Amblyomin-X blocked NFKB1 cleavage after proteasome inhibition, suggesting the inhibition of the NF-κB complex; thus, dynein function could not be related to its translocation to the nucleus. The last finding is also present in proteasome inhibitors such as bortezomib [32].

We found that Amblyomin-X possibly induced aggresome formation via the non-exclusive ubiquitin pathway because Bag3 levels were increased. This is in contrast to other proteasome inhibitors that form aggresomes through the exclusive ubiquitin pathway [32]. Additionally, aggresomes were formed in a nascent polypeptide-dependent manner after the recombinant protein induction; this could be concluded because CHX pretreatment abolished the aggresome formation induced by Amblyomin-X. The explanation for this involves the reduction of proteasome cargo by inhibiting protein synthesis on ribosomes to a level that is not sufficient to form aggresomes, as previously reported [40]. This result also occurs with proteasome inhibitors such as bortezomib [40].

Moreover, transmission electron microscopy revealed spherical-type aggresome formation in MIA PaCa-2 cells and ribbon-type aggresome formation in SK-MEL-28 cells. The two aggresome types can be composed of different substrates, such as multispanning transmembrane proteins, secretory proteins and cytosolic proteins. The structure varies depending on the aggregating proteins and the cell type [11]. The melanoma cell line (SK-MEL-28) represents malignant cells derived from melanocytes localized in the epithelial tissue of skin. The pancreas adenocarcinoma cell line (MIA PaCa-2) represents malignant cells localized in the pancreas, a gland of the digestive and endocrine systems. We hypothesize that the aggresomes that formed in MIA PaCa-2 cells had secretory proteins because these proteins are related to pancreas function. These proteins could represent a relevant factor in the formation of the spherical-type (as opposed to ribbon-type aggresomes) in this cell line.

Surprisingly, aggresomes were not cleared by autophagy, as is expected for a proteasome inhibitor [32]. The conversion of LC3-I into LC3-II was not observed, which could lead to an enhanced activity of the recombinant protein. Furthermore, we did not observe increased acidic vesicle formation or autophagic vacuoles after Amblyomin-X treatment, indicating no increased formation of late endosomes or lysosomes. Additionally, pretreatment with rapamycin followed by Amblyomin-X returned acidic vesicles to basal levels, possibly indicating a possible role for mTOR in the inhibition of autophagy activation. To test this hypothesis, we measured mTOR and its co-localization with HC1, once dynein was reported to transports mTOR to perform its function [44]. Indeed, Amblyomin-X induced the overexpression of mTOR, which co-localized with dynein.

The light chains LC8-1 and LC8-2 of dynein can function with or without being coupled to the molecular motor complex [38], [43]. LC8-2, in particular can be involved in apoptosis [38], [43] or autophagy [39], [45]. The increases observed in the mRNA and protein levels of LC8-2 induced by Amblyomin-X in tumor cells together with the decreased expression of AMBRA1 and its co-localization with dynein chains only in MIA PaCa-2 cells suggest that AMBRA1 could be sequestered to LC8-2 to become inactive. Although the co-localization of both targets did not occur in SK-MEL-28 cells, the decreased AMBRA1 suggests that Amblyomin-X inhibited autophagy activation. The pro-apoptotic factor Bim, which can participate in both apoptosis and autophagy (assisted by dynein) [38], [39], [43], [45] appears to perform none of these biological functions in this mechanism of action and thus does not influence the inhibition of autophagy activation or the programmed cell death triggered by the recombinant protein in the studied tumor cells.

This work demonstrated that Amblyomin-X induces a series of intracellular events that are cytotoxic for tumor cells and lead to tumor cell death. The events observed by the presence of Amblyomin-X in tumor cells differentiate this molecule from other proteasome inhibitors. These differences rely on the following: (i) Amblyomin-X appears to inhibit the proteasome and to inhibit autophagy activation, thus influencing more than one target and then enhancing the therapeutic potential of the molecule; (ii) Amblyomin-X appears to be a non-cell-permeable molecule; (iii) Amblyomin-X is a protein and not a small molecule; (iv) Amblyomin-X induces aggresome formation possibly via the non-exclusive ubiquitin pathway; (v) Amblyomin-X requires dynein involvement and altered gene and protein expression; (vi) Amblyomin-X preferentially inhibits the trypsin-like activity of the proteasome. These findings provide new insights into the molecular mechanism of action of Amblyomin-X, suggesting dynein as a possible new target. The recombinant protein is a promising molecular entity to treat malignant tumors and does not act as a classic proteasome inhibitor because it can affect more than one target.
